# Mr. Headly, on Angina Maligna

**Published:** 1801-05

**Authors:** Henry Headly

**Affiliations:** Calne, Wilts


### *To the Editors of the Medical and Phyſical Journal.*

Gentlemen,

THE Angina Maligna being a diſeaſe which has made its appearance in this kingdom, with more or leſs ſeverity for ſeveral years; and it being probable it may continue to viſit us, I beg leave to recommend a plan for preventing the ſpreading of the contagious effluvia; a plan which I adopted in the year 1791, and have ſince witneſſed its ſucceſs in ſome hundreds of caſes.

It would be ſuperfluous in me to give a full hiſtory of this diſeaſe, as we have it from a more able man, Dr. Fothergill, who wrote in the year 1748. This author, after noticing the opinions and practice of the Spaniſh and Italian writers on the ſubject, gives a very faithful and perfect detail of its firſt appearance in this country; alſo its progreſs, with the mode of cure. He concludes his treatiſe, by obſerving,

1ſt. That the ſore throat, attended with ulcers, ſeems to be accompanied with a ſtrong diſpoſition to putrefaction, which affects the habit in general; but the fauces, and the parts contiguous, in particular; and it ſeems not unreaſonable to ſuppoſe,

2dly. That the cauſe of this tendency is a putrid virus, or miaſma ſui generis, introduced into the habit by contagion, principally by means of the breath of the perſon affected.

3dly. That this virus, or contagious matter, produces effects more or leſs pernicious, according to the quantity and nature of the infection, and as the ſubject is diſpoſed to receive or ſuffer by it.

4thly. That putrefactive and malignant diſeaſes, in common, admit of the moſt ſenſible and ſecure relief from diſcharges of the peccant matter, either upon the ſkin in general, or on particular parts of the body.

5thly. That the redneſs and cutaneous efflereſcence in the preſent caſe, may be conſidered as an eruption of the like nature, and therefore to be promoted by ſuch methods as have proved ſucceſsful in ſimilar caſes.

As my paper offers only a recommendatory plan of prevention, it may ſeem a digreſſion in my noticing a curative caution, as to the mode of treating the ulcers; but this I am prompted to, from obſerving the attention (recommended by Spaniſh authors, as well as by gentlemen in the iſland of St. Vincent and Grenada), to the uſe of local applications to the throat. In two letters from John Collins, Eſq. of the Iſland of St. Vincent, cent, addreſſed to Benjamin Vaughan, Eſq. of London, in the year 1790, is deſcribed a diſeaſe not confining itſelf to the above iſlands, but a diſeaſe that extended through the whole range of iſlands in that quarter; its attacks were followed with fatality in a great many caſes. Bark and tonic medicines were given with little or no effect; and the gangrenous diſpoſition of the throat was only checked by capſicum; a remedy which at once gives us not only the evident ſenſe of its activity, but the ample proof of its innocence, when taken in a large quantity; a remedy which is uſed in intermittent fevers, and for the ſuppreſſion of vomitings, in the putrid fevers of warm countries, with ſucceſs.

But to the point. My own obſervations lead me to obſerve, that the diſeaſe in queſtion generally makes its firſt attack on weakly children; that its conſequences depend upon ſeaſons, ſituations, conſtitution, and method of treatment; that in one or two caſes, at firſt, it may prove of a benign and doubtful nature; but that the efflorefcence on the ſkin will expoſe to us the inſidious enemy; that it is communicated by the contagious effluvia from an ulcerated throat lodging on the tonſils of other perſons; that death to a certain extent is produced in the part; that no inconvenience is at firſt felt, more than an aching, a ſtiffneſs, and an inability to move the neck; that in a day or two the ſlough is removed by ulcerative inflammation; that vomiting, chillineſs, and fever, with head-ach, now come on, accompanied by heart-burn and diarrhœa; that theſe ſymptoms are ſucceeded by an efflorefcence on the ſkin, and by perſpiration, which generally prove ſalutary, and lead to a cure. Having related what has appeared to me the uſual courſe of the diſeaſe, I will briefly mention my method of prevention. It conſiſts, in ordering to all the attendants of the ſick, a tea ſpoonful or two of the under written gargle to be uſed, and ſwallowed every two hours. It is meant to produce and keep up a regular excitement on the tonſils, uvula, and fauces; and by that means enable them to reſiſt the ſedative effects of the human poiſon. I will repeat what I before mentioned, that where the gargle is ſufficiently pungent, and often enough uſed, I have never ſeen it fail.

℞. Pip. Cayenn. coch. j. magn. Sal. comm. coch. j. min. aq. bull. ℥iij. acet. diſtill. ℥iij. f. garg.

I am, &c.

*Calne, Wilts, March* 16, 1801. HENRY HEADLY.

